# Assessing the Impact of the COVID-19 Pandemic on Pediatric Emergency Department Visits in Taiwan

**DOI:** 10.3390/medicina60020288

**Published:** 2024-02-08

**Authors:** Yu-Ting Lee, Yen-Wen Lai, Jiann-Hwa Chen, Wei-Lung Chen, Meng-Yu Wu, Jui-Yuan Chung

**Affiliations:** 1 Department of Emergency Medicine, Cathay General Hospital, Taipei 106438, Taiwan; j79312@hotmail.com (Y.-T.L.); chenjiannhwas@yahoo.com.tw (J.-H.C.); weilung.chen@msa.hinet.net (W.-L.C.); 2 Department of Emergency Medicine, Sijhih Cathay General Hospital, New Taipei City 221037, Taiwan; f08942002@gmail.com; 3 School of Medicine, Fu Jen Catholic University, Taipei 242062, Taiwan; 4 Department of Emergency Medicine, Tzu Chi Hospital, Buddhist Tzu Chi Medical Foundation, New Taipei City 231016, Taiwan; 5 School of Medicine, Tzu Chi University, Hualien 970374, Taiwan; 6 School of Medicine, National Tsing Hua University, Hsinchu 300044, Taiwan; 7 Department of Education, Cathay General Hospital, Taipei 106438, Taiwan

**Keywords:** COVID-19, pandemic, pediatric, emergency department visits

## Abstract

*Background and Objectives*: The coronavirus disease 2019 (COVID-19) pandemic has profoundly impacted healthcare systems worldwide. To assess the effects of the pandemic on pediatric emergency department (ED) visits in Taiwan, we conducted a study to evaluate changes in pediatric ED visits during the COVID-19 pandemic. *Materials and Methods*: This retrospective study included pediatric patients (age ≤ 18) who visited the ED between 21 January 2019 and 30 April 2019, at three hospitals of the Cathay Health System, and compared them with a corresponding period in 2020. Basic information, including mode of arrival, triage level, disposition, chief complaints, and incidence rates, were analyzed before and during the pandemic. *Results*: A total of 10,116 patients, with 6009 in the pre-pandemic group and 4107 in the pandemic group, were included in this study. The mean number of daily pediatric ED visits decreased from 60.09 before the pandemic to 40.66 during the pandemic, while ambulance use increased significantly by 2.56%. The percentage of patients with high acuity triage levels (levels 1 and 2) was significantly lower during the pandemic period (0.63% and 10.18%, respectively) than the pre-pandemic period (0.7% and 10.9%, respectively). Additionally, a significantly higher proportion of patients were discharged during the pandemic period (89.36%) than during the pre-pandemic period (88.33%). The proportion of COVID-19-related complaints, such as fever and respiratory tract infections, as well as other complaints including gastrointestinal issues, trauma, and psychological problems, significantly increased during the pandemic. *Conclusions*: In preparation for future pandemics, we recommend increasing emergency medical service capacity, establishing a non-contagious route for obtaining chronic medication prescriptions, optimizing staff allocation in pediatric emergency departments, and increasing the number of hospital social workers for enhanced support.

## 1. Introduction

Coronavirus disease 2019 (COVID-19) is a highly contagious novel virus that has caused countless deaths worldwide since its outbreak at the end of 2019. The virus spreads rapidly via droplet transmission and can travel up to one meter. The incubation period is up to 14 days, with most patients showing symptoms after 5–6 days. Common symptoms include cough, rhinorrhea, fever, and nasal congestion. Some patients develop pneumonia and respiratory failure [1].

Taiwan announced its first imported case of COVID-19 on 21 January 2020. The first in-hospital COVID-19 cluster infection occurred at the end of February 2020, marking the beginning of the peak epidemic period [2,3]. From March to April 2020, the number of confirmed COVID-19 cases in Taiwan increased rapidly, with 759 confirmed cases as of 19 December 2020.

Although some countries have experienced overcrowding in their healthcare systems, data from other countries have shown a decrease in the number of ED patients; according to a study conducted in the United States, ED visits declined by 45.7% in 2020 compared to the preceding years, 2017 to 2019 [4,5]. The pandemic has resulted in significant changes in ED visiting habits, including a decrease in visits for non-COVID-19 diseases and an increase in visits for out-of-hospital cardiac arrest (likely due to delays in seeking medical care) [6]. These changes in ED visiting habits, along with the use of emergency medical services, triage levels, and chief complaints, play a crucial role in evaluating and redistributing medical resources during the epidemic season.

The global impact of the COVID-19 pandemic on pediatric healthcare, especially within emergency departments (EDs), has been substantial [7]. Pediatric healthcare institutions worldwide may need to reallocate resources to address the surge in COVID-19 patients. This may entail expanding pediatric bed capacity, reinforcing intensive care units to manage severe cases, and adapting human resources to meet evolving demands. Additionally, in a bid to minimize virus transmission, certain regions have increased the provision of remote pediatric healthcare services. This encompasses remote diagnosis, online consultations, and remote monitoring, ensuring that patients can access essential medical care while avoiding gatherings within healthcare facilities [8]. Beyond the physical implications, the pandemic has taken a toll on the mental health of children, exposing them to isolation, changes in learning environments, and the stress associated with the spread of the disease [9]. Consequently, healthcare institutions may find it necessary to provide additional psychological support and resources in response.

Despite the significant impact of the COVID-19 pandemic on healthcare systems worldwide, there is a lack of research investigating its effects on pediatric emergency department (ED) visits. Therefore, this study aims to assess the impact of the pandemic on various aspects of pediatric ED visits, including patient volume, mode of arrival, triage level, disposition, time of visit, and chief complaints. These findings may help healthcare providers allocate medical resources more effectively and avoid the potential overcrowding of the healthcare system.

## 2. Methods

### 2.1. Study Setting

This retrospective study was conducted in three hospitals within the Cathay Health system in Taiwan. The study recruited patients who visited the ED of these hospitals between 21 January 2020 and 30 April 2020, as well as patients who visited between 21 January 2019 and 30 April 2019. One of the hospitals was a tertiary center located in Taipei City, with a capacity of 800 beds and an estimated total annual ED visit volume of 60,000. The other two hospitals were located in rural areas and had capacities of 642 and 348 beds, with an estimated annual ED visit volume of 48,000 and 30,000, respectively.

### 2.2. Study Design

**Figure 1:**
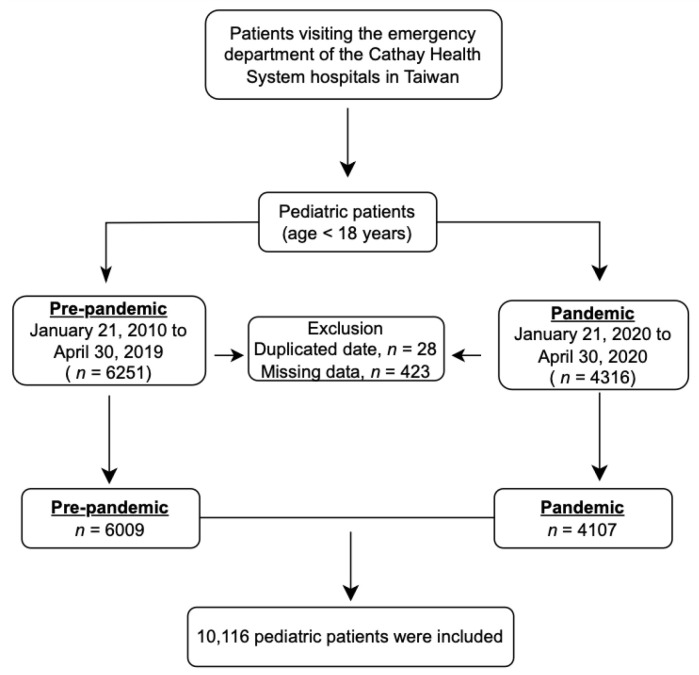
Flowchart of this study.

Patient information was extracted from the electronic medical record (EMR) system and divided into two groups: the “during epidemic” group (21 January 2020 to 30 April 2020) and the “before epidemic” group (21 January 2019 to 30 April 2019). The epidemic period was defined as the time between the first confirmed case of COVID-19 in Taiwan on 21 January 2020 and the end of the period on 30 April 2020, which was the fourth day after no confirmed cases were reported for three consecutive days. All patients, regardless of age, who presented at the EDs during these two periods were considered eligible for recruitment. Patients with missing and duplicate data were excluded. The flowchart depicting the key steps of this study is presented in Figure 1.

### 2.3. Variables

Basic demographics, visit characteristics, dispositions, and chief complaints of patients visiting the ED during the two periods were obtained from the electronic medical record (EMR) system. Visit characteristics, such as total daily visits, age, sex, mode of arrival, triage, and disposition were recorded, while chief complaints were extracted from patients’ narratives recorded in the EMR and sorted into 33 common discomforts. Patients were triaged based on a combination of the Canadian Triage and Acuity Scale (CTAS) and clinical criteria. Triage nurses with formal training and more than one year of ED working experience assigned patients to different triage levels.

### 2.4. Ethical Statement

The Institutional Review Board of the Cathay General Hospital Bioethical Committee approved the study, conducted in adherence to the Declaration of Helsinki (approval number: CGH-P109047). It was an observational study without any additional interventions beyond standard patient care. Consequently, the need for informed consent was waived by the Institutional Review Board of the Cathay General Hospital Bioethical Committee.

### 2.5. Statistical Analysis

We analyzed the data using IBM SPSS Statistics for Windows (version 25.0). Categorical variables are presented as numbers and percentages, while normally distributed continuous variables are presented as mean ± standard deviation (SD). We used the chi-square test to analyze categorical variables and an independent *t*-test to analyze normally distributed continuous variables. Incidence rates were calculated by dividing the number of results of interest by the total number of ED visits during each period. The incidence rate ratio (IRR) was calculated by dividing the incidence rate during the epidemic period by that before the epidemic period. Further, we computed the percentage difference in the number of chief complaints between the “before epidemic” and “during epidemic” periods.

## 3. Results

A total of 10,116 children were included in this study. There were 6009 ED visits before the pandemic (21 January 2019 to 30 April 2019) (Figure 1). During the pandemic period, there was a reduction of 31.65% (4107 ED visits) compared to the pre-pandemic period (Table 1). Furthermore, the mean number of daily pediatric ED visits (total pediatric ED visits divided by the total days of the study period) decreased, from 60.09 before the pandemic to 40.66 during the pandemic. Although most of the characteristics had decreased numbers during the pandemic, compared to the pre-pandemic period, a significantly higher proportion of female pediatric ED patients was exhibited during the pandemic (43.14 vs. 43.39).

The utilization proportion of the emergency medical service (EMS) system (ambulance) exhibited a notable increase during the pandemic period (5.97%) compared to the pre-pandemic period (3.41%). The percentage of patients with high acuity level, triage levels 1 and 2, was significantly lower during the pandemic period (0.63% and 10.18%, respectively) than during the pre-pandemic period (0.7% and 10.9%, respectively). On the other hand, the percentage of patients with low acuity level, triage levels 3 and 4, was significant higher during the pandemic period (82.08% and 6.60%, respectively) than during the pre-pandemic period (81.53% and 6.17%, respectively). Additionally, there was a significantly higher proportion of patients discharged during the pandemic period (89.36%) than during the pre-pandemic period (88.33%) (Table 1).

The incidence rates of pediatric ED visits for infection-associated issues, such as fever and upper respiratory infection (URI) symptoms, were 0.90 and 0.89 times lower during the pandemic period, respectively (*p* < 0.01). Gastrointestinal-associated issues, such as abdominal pain and acute gastroenteritis (AGE) symptoms, had IRR levels of 0.95 and 0.79 during the pandemic period, respectively (*p* < 0.01).

Regarding cardiovascular-associated issues, pediatric ED patients with chief complaints of hypertension were 16.33 times higher during the pandemic period. The incidence of neurology-related complaints, particular headache and convulsion, were 0.65 and 0.35 times lower during the pandemic compared to the pre-pandemic period (*p* < 0.01). Pediatric patients visiting the ED with chief complaints of trauma and psychological problems had IRR levels of 1.01 and 4.60, respectively, while patients with chief complaints of facial feature problems were 0.72 times lower in the pandemic period compared to the pandemic period (*p* < 0.01) (Table 2).

Increased IRR was also observed in the category of other visiting chief complaints for malaise and myalgia (IRR = 1.35 and 2.02, respectively), shortness of breath (IRR = 1.26), glycemic problems (IRR = 5.4), urological symptoms (IRR = 1.09), and social problems (IRR = 1.46), as well as critical complaints, including cardiac arrest (IRR = 5) and altered mental status (IRR = 3.08), during the pandemic (Table 2), despite the absence of statistical significance.

## 4. Discussion

Numerous studies have demonstrated a significant decrease in ED visits across almost all age groups during the early phase of the pandemic [4,5,10,11]. However, contrary to the increased proportion of adults visiting the ED, from 60.5% (pre-pandemic) to 65.2% (pandemic) [11], our research revealed a decreased proportion and number of pediatric ED visits across all Cathay Health System hospitals during the pandemic season. Several factors, such as school closures, social distancing policies, and media influence, could have contributed to the decline in total pediatric ED visits [7,8]. This phenomenon is particularly evident among vulnerable populations, such as pediatric patients, as parents and caregivers were often hesitant to bring their children to the hospital due to fear of COVID-19 infection during ED visits.

Based on our research, we identified several impacts of the COVID-19 pandemic on our pediatric emergency department (PED). We noted a decrease in the number of children arriving being held or walking, whereas the percentage of ambulance use increased from 3.41% to 5.97%. Additionally, the percentage of patients with low triage acuity levels (levels 3 and 4) was higher during the pandemic period (88.68%) than during the pre-pandemic period (87.7%). This may explain the increased proportion of pediatric patients who were discharged during the pandemic period (88.33% to 89.36%). Furthermore, we observed a decrease in the percentage of admissions, from 10.65% to 9.89%. However, we also noted an increased percentage of ambulance use during the pandemic period, indicating that parents tended to call for emergency medical attention for their children, even for mild symptoms, because of the fear of infection.

In contrast to our study, research from the United States of America reported a decrease in PED visits and an increase in admission rates during the early phase of the pandemic period [12]. In addition, a study from Finland revealed a decline in the number of EMS missions within the first two months after the first COVID-19 cases in the study area [13]. The contradictory results from the two studies may be due to differences in local healthcare-seeking behaviors. Taiwanese people have access to convenient medical resources but may be afraid of severe disease complications, especially given the sensationalized news on COVID-19 on social media. Parents may be anxious about their children’s health but hesitant to take them to hospital. Consequently, they may opt to call an ambulance, even for minor illnesses. If the doctor’s examination reveals no significant health issues, they may opt for medication instead of hospitalization.

Second, our study revealed a higher percentage of pediatric trauma cases during the pre-pandemic and pandemic periods (23.31% and 23.62%, respectively). However, several studies have documented a decrease in the percentage of pediatric trauma cases during the pandemic period, likely attributed to reduced social activities and traffic mobility resulting from the enforcement of “stay-at-home” policies [14,15,16,17]. We thought the differences between these results may be due to the convenience of seeking medical aid in Taiwan, where parents might bring their children to the emergency room for even minor injuries. Notably, there has been a shift in the mechanisms of injury, evidenced by an increase in home-related penetrating injuries [16]. Pediatric patients who visited emergency departments during the pandemic period presented with more severe injuries, necessitating extended stays in the Intensive Care Unit (ICU). Overall, through these studies, we found that the incidence of trauma decreased, but home-related injuries such as penetrating wounds increased, as did the severity of trauma. Regardless of the results, despite children spending more time at home during quarantine, vigilance regarding the occurrence of home accidents remains crucial [17].

Third, although there was no significant difference, life-threatening chief complaints, including altered mental status and cardiac arrest, demonstrated IRR levels of 3.08 and 5.00, respectively. Our findings align with similar studies conducted in other countries (UK and Singapore), which also reported an increased incidence of out-of-hospital cardiac arrest and worse outcomes during the pandemic [18,19]. Additionally, we observed an increase in the IRRs of the chief complaints of shortness of breath, hypertension, and glycemic problems (1.28, 16.33, and 5.4, respectively). A prospective multicenter study reported that children with COVID-19 have significantly higher systolic and diastolic blood pressure than healthy children [20]. Moreover, a study from India found that glycemic control in type 1 diabetes mellitus patients worsened during the pandemic period, although the study mainly focused on adults [21]. Pediatric patients with asthma and acute exacerbation often present to the ED with the chief complaint of wheezing (indicated by shortness of breath) [22]. Long-term medication, such as bronchodilators, antihypertensive drugs, insulin, and oral antidiabetic agents, are necessary to control these chronic diseases. During the pandemic, obtaining these long-term medications may have been difficult due to lockdown policies, reduced outpatient appointments at local clinics or hospitals, and the fear of infection. Therefore, during a pandemic, it is crucial to prepare more backup, long-term medications for the public or increase online pharmacy services for medication pickup.

An interesting finding of our study was the decreased percentage of chief complaints related to headaches and convulsions. A significant proportion of headaches in children are caused by fever, which can be associated with upper respiratory tract infections. Additionally, febrile seizures are a common cause of pediatric convulsions [23]. According to our research, the percentage of chief pediatric complaints of fever and upper respiratory tract infections decreased during the pandemic period, which may be attributed to the lockdown strategy and health education policies, such as mask-wearing and improved hygiene practices. This finding is consistent with a study from Saudi Arabia, which also found a decrease in visits for common neurological conditions, such as headache and seizures, during the pandemic period [24]. A research study conducted in New York City reported a 64% reduction in visits to the pediatric ED concerning headaches during the pandemic [25]. Likewise, another multicenter study reported a 28% decrease in ED visits for seizures during the same period [26]. These declines in ED visits for neurological conditions were associated with various factors, including shifts in healthcare-seeking behavior influenced by the pandemic, concerns about contracting COVID-19, and alterations in daily routines and activities.

Finally, an increased proportion of children presenting with social problems, such as domestic violence, were noted during the pandemic compared with the pre-pandemic period in our study. The implementation of stay-at-home orders has been associated with a notable surge in domestic violence incidents [27]. This increase is likely attributable to various factors, including elevated unemployment rates, heightened stress related to childcare and homeschooling, and the economic impacts stemming from the pandemic [28,29]. Consequently, for many families experiencing domestic violence, home may not be a safe environment. This vulnerability extends to both intimate partners and children. We found some studies indicated that the “stay-at-home” policy during the pandemic period may have led to an increase in domestic violence [27,28,29]. A systematic review, encompassing literature from North America, Europe, Asia-Pacific, and Africa, highlighted the impact of COVID-19 on domestic violence cases. The findings indicated a notable increase, particularly during the initial week of lockdowns imposed in various countries [28]. Another systemic review and meta-analysis also revealed that incidents of domestic violence increased in response to “stay-at-home/lockdown” strategies [29]. While it has always been traditionally recognized that “home is a shelter for children”, the confinement measures resulted in continuous contact between perpetrators and victims, leading to an escalation in violent behavior. Addressing such issues necessitates the implementation of preventive measures and supportive programs. We can develop corresponding policies, such as strengthening the Social Welfare Bureau’s concern for high-risk families and promoting telephone hotlines to provide victims with access to appeal and psychosocial support.

Additionally, this study indicates a significant increase in the proportion of psychological problems during the pandemic. According to the Centers for Disease Control and Prevention data in the US, the pandemic has been associated with an increase in ED visits for mental-health-related issues among children, with a 24% increase for children aged 5 to 11 and a 31% increase for those aged 12 to 17 [30]. The strain on pediatric mental health resources has been attributed to various factors, including the stress of lockdowns, loss of caregivers, and school interruptions [31]. We also found that one study revealed that the rates of suicidal ideation and attempts were higher during the pandemic period [32]. A meta-analysis study revealed that COVID-19 is associated with an increase in suicidal behavior, including elevated event rates for suicidal ideation, suicide attempts, and self-harm. Specifically, their results indicate that individuals who are younger, female, and from democratic countries were most susceptible to suicidal ideation during the COVID-19 pandemic [9]. These results are consistent with our studies and the possible reasons may stem from social isolation, anxiety, fear of contagion, uncertainty, chronic stress, and economic difficulties [33]. Therefore, more social workers should be recruited in the ED in response to these circumstances.

## 5. Limitation

Our study has several limitations. First, this was a retrospective study that relied on EMR data from 2019 to 2020, which may have resulted in some missing data. Second, all three hospitals included in the study are located in northern Taiwan, which may limit the generalizability of our findings to other regions of the country. Third, the number of patients in certain subgroups of chief pediatric complaints was small, which may limit the generalizability of our findings to the pandemic period. Lastly, although all triage nurses underwent formal training, they may have assigned different emergency severity indexes (ESIs) to different patients according to their preferences.

## 6. Conclusions

In preparation for potential future pandemics, we propose the following recommendations: Increase the capacity of EMS and enhance prehospital emergency medical services. Establish a non-contagious route for obtaining prescriptions for chronic medication. Given the study’s findings, showing a decrease in the proportion of minor pediatric chief complaints during the pandemic, there is a need to optimize the allocation of staff in pediatric EDs. The number of hospital social workers should be augmented to address the increased demand for mental health care.

## Figures and Tables

**Table 1:** Comparison of the characteristics of pediatric patients’ emergency department visits before and during the epidemic.

Characteristics	Before Epidemic (January 2019~April 2019) (*n* = 6009)	During Epidemic (January 2020~April 2020) (*n* = 4107)	Difference (%)	*p*-Value
Total visits/day— (mean ± SD)	60.09 ± 20.5	40.66 ± 23.6	−31.65	<0.01
Age—*n*. (%)				
12 ≤ Age < 18	1388 (23.10)	1462 (35.60)	+5.33	0.57
Age < 12	4621 (76.90)	2645 (64.40)	−42.76	<0.01
Sex—no. (%)				
Male	3417 (56.86)	2325 (56.61)	−31.96	<0.01
Female	2592 (43.14)	1782 (43.39)	−31.25	<0.01
Mode of arrival—*n*. (%)				
Walk-in	3939 (65.56)	2796 (68.08)	−29.02	<0.01
Carried	1511 (25.14)	675 (16.44)	−55.33	<0.01
Ambulance	205 (3.41)	245 (5.97)	+19.51	<0.01
Others	354 (5.89)	391 (9.52)	−9.46	0.31
Triage—*n*. (%)				
Triage 1	42 (0.70)	26 (0.63)	−38.10	0.04
Triage 2	655 (10.90)	418 (10.18)	−36.18	<0.01
Triage 3	4899 (81.53)	3371 (82.08)	−31.19	<0.01
Triage 4	370 (6.17)	271 (6.60)	−26.76	<0.01
Triage 5	43 (0.70)	21 (0.51)	−51.16	<0.01
Disposition—*n*. (%)				
Admission	640 (10.65)	406 (9.89)	−36.56	<0.01
Discharge	5308 (88.33)	3670 (89.36)	−30.86	<0.01
AMA	47 (0.78)	18 (0.44)	−61.70	<0.01
Transfer	12 (0.20)	10 (0.24)	−16.67	0.69
Mortality	2 (0.04)	3 (0.07)	+50.00	0.65

SD, standard deviation; AMA, against medical advice.

**Table 2:** Comparison of chief complaints in pediatric patients’ emergency department visits before and during the COVID-19 epidemic.

Chief Complaints	Before Pandemic (January 2019~March 2019)		During Pandemic (January 2020~March 2020)		Difference (%)	Incidence Rate Ratio	*p*-Value
	*n*	Incidence (%)	*n*	Incidence (%)			
Infection-related complaints							
Fever	2033	33.83	1254	30.53	−38.32%	0.90	<0.01
URI	1148	19.10	697	16.97	−39.29%	0.89	<0.01
Cellulitis	75	1.25	64	1.56	−14.67%	1.25	0.28
Gastrointestinal-related complaints							
Abdominal pain	689	11.47	445	10.84	−35.41%	0.95	<0.01
AGE symptoms	489	8.14	264	6.43	−46.01%	0.79	<0.01
Constipation	20	0.33	11	0.27	−45.00%	0.82	0.09
GI bleeding symptoms	11	0.18	15	0.37	36.36%	2.06	0.55
Cardiovascular-related complaints							
Chest pain	52	0.87	98	2.39	88.46%	2.75	0.01
Hypertension	2	0.03	20	0.49	900.00%	16.33	<0.01
Neurology-related complaints							
Dizziness	107	1.78	96	2.34	−10.28%	1.32	0.46
Headache	118	1.96	52	1.27	−55.93%	0.65	<0.01
Convulsion	50	0.83	29	0.71	−42.00%	0.35	<0.01
Altered mental status	7	0.12	15	0.37	114.29%	3.08	0.10
Malaise	12	0.20	11	0.27	−8.33%	1.35	0.83
Myalgia	65	0.01	83	2.02	27.69%	202.00	0.65
Shortness of breath	155	2.58	135	3.29	−12.90%	1.26	0.28
Glycemic problems	3	0.05	11	0.27	266.67%	5.40	0.07
Urological symptoms	78	1.30	58	1.41	−25.64%	1.09	0.12
Trauma	1401	23.31	970	23.62	−30.76%	1.01	<0.01
Facial feature problems ^+^	217	3.61	106	2.58	−51.15%	0.72	<0.01
Cardiac arrest	2	0.03	6	0.15	200%	5.00	0.31
Psychological problems	6	0.10	19	0.46	216.67%	4.60	0.03
Social problems *	13	0.22	13	0.32	0%	1.46	0.71
Others	49	0.82	18	0.44	−63.27%	0.54	<0.01

URI, upper respiratory infection; AGE, acute gastroenteritis; GI, gastrointestinal. ^+^ Eye, ENT, and dental problems * Family violence and sexual assault.

## Data Availability

The datasets used and/or analyzed regarding to the current study are available from the corresponding author on reasonable request.

## Abbreviations

COVID-19	coronavirus disease 2019
ED	emergency department
IRR	incidence rate ratio
EMR	electronic medical record
CTAS	Canadian Triage and Acuity Scale
SD	standard deviation
URI	upper respiratory infection
AGE	acute gastroenteritis
GI	gastrointestinal
PED	pediatric emergency department
ESIs	emergency severity indexes
EMS	emergency medical service
